# Non-contrast MRI protocol for TAVI guidance: quiescent-interval single-shot angiography in comparison with contrast-enhanced CT

**DOI:** 10.1007/s00330-020-06832-7

**Published:** 2020-04-22

**Authors:** Mathias Pamminger, Gert Klug, Christof Kranewitter, Martin Reindl, Sebastian J. Reinstadler, Benjamin Henninger, Christina Tiller, Magdalena Holzknecht, Christian Kremser, Axel Bauer, Werner Jaschke, Bernhard Metzler, Agnes Mayr

**Affiliations:** 1 University Clinic of Radiology, Medical University of Innsbruck, Anichstraße 35, A-6020 Innsbruck, Austria; 2 University Clinic of Internal Medicine III, Cardiology and Angiology, Medical University of Innsbruck, Anichstraße 35, A-6020 Innsbruck, Austria

**Keywords:** Transcatheter aortic valve implantation, CT angiography, Cardiac imaging technique, Contrast media, Renal insufficiency

## Abstract

**Objectives:**

To prospectively compare unenhanced quiescent-interval single-shot MR angiography (QISS-MRA) with contrast-enhanced computed tomography angiography (CTA) for contrast-free guidance in transcatheter aortic valve intervention (TAVI).

**Methods:**

Twenty-six patients (mean age 83 ± 5 years, 15 female [58%]) referred for TAVI evaluation underwent QISS-MRA for aortoiliofemoral access guidance and non-contrast three-dimensional (3D) “whole heart” MRI for prosthesis sizing on a 1.5-T system. Contrast-enhanced CTA was performed as imaging gold standard for TAVI planning. Image quality was assessed by a 4-point Likert scale; continuous MRA and CTA measurements were compared with regression and Bland-Altman analyses.

**Results:**

QISS-MRA and CTA-based measurements of aortoiliofemoral vessel diameters correlated moderately to very strong (*r* = 0.572 to 0.851, all *p* ≤ 0.002) with good to excellent inter-observer reliability (intra-class correlation coefficient (ICC) = 0.862 to 0.999, all *p* < 0.0001) regarding QISS assessment. Mean diameters of the infrarenal aorta and iliofemoral vessels differed significantly (bias 0.37 to 0.98 mm, *p* = 0.041 to < 0.0001) between the two modalities. However, inter-method decision for transfemoral access route was comparable (*κ* = 0.866, *p* < 0.0001). Aortic root parameters assessed by 3D whole heart MRI strongly correlated (*r* = 0.679 to 0.887, all *p* ≤ 0.0001) to CTA measurements.

**Conclusion:**

QISS-MRA provides contrast-free access route evaluation in TAVI patients with moderate to strong correlations compared with CTA and substantial inter-observer agreement. Despite some significant differences in minimal vessel diameters, inter-method agreement for transfemoral accessibility is strong. Combination with 3D whole heart MRI facilitates unenhanced TAVI guidance.

**Key Points:**

*• QISS-MRA and CTA inter-method agreement for transfemoral approach is strong.*

*• QISS-MRA is a very good alternative to CTA and MRA especially in patients with Kidney Disease Outcomes Quality Initiativestages 4 and 5.*

*• Combination of QISS-MRA and 3D “whole heart” MRI facilitates fully unenhanced TAVI guidance.*

## Introduction

The standard pre-imaging workup of transcatheter aortic valve intervention (TAVI) currently consists of a combination of transthoracic and transesophageal echocardiography as well as contrast-enhanced aortoiliacal CTA for the exact determination of valve size and implantation route [1]. However, the administration of a contrast agent may be hindered by acute or chronic kidney disease (CKD), with reported prevalence rates up to 41% for acute kidney injuries and up to 70% for CKD in patients undergoing TAVI assessment [2, 3]. Therefore, cross-sectional imaging techniques to reduce patient exposure to iodinated contrast agents are desirable [4]. Our study group recently presented a contrast-enhanced MRI protocol to provide measurements of aortic annulus and access routes with good to excellent agreement in comparison with CTA resulting in accordant decisions for prosthesis size as well as transfemoral TAVI access capability [5]. However, in patients with stage 4 and 5 renal impairment (estimated glomerular filtration rate (eGFR) < 30 ml/min/1.73 m^2^), rare cases of nephrogenic systemic fibrosis (NSF), a severe and potentially fatal condition without known effective treatment, were reported [6]. Moreover, recent reports of intracranial gadolinium deposition with currently unclear clinical relevance have fostered efforts to develop unenhanced MRI protocols [7, 8].

The QISS technique represents a clinically feasible and efficient non-contrast MRA, however, until now mainly evaluated in peripheral arterial disease (PAD) [8–10]. Hence, we sought to compare a comprehensive non-contrast-enhanced MRI protocol, including three-dimensional (3D) “whole heart” acquisition and QISS-MRA with CTA for TAVI planning using aortic root and access vessel measurements as well as decision for transfemoral accessibility.

## Material and methods

Twenty-six patients (mean age 83 ± 5 years) referred for TAVI evaluation underwent non-contrast MRI and additional CTA within 1 day (interquartile range (IQR) 0–2 days). In addition to the 26 patients who received both MRI and CTA examinations, nine additional patients underwent a MRI scan exclusively. This results in a MRI cohort of a total of 35 patients for inter-observer comparison and image quality evaluation of QISS-MRA.

Local ethics committee approval was provided prior to inclusion of the first patient and written informed consent was obtained from each participant.

General inclusion criteria were severe aortic stenosis according to recent guidelines (aortic valve area ≤ 1.0 cm^2^ or aortic valve index ≤ 0.6 cm^2^/m^2^) with the decision for TAVI procedure by an interdisciplinary heart team and typical symptoms of severe aortic stenosis like shortness of breath, angina, or syncope [11, 12].

Exclusion criteria were contraindications to perform MRI or CTA; contraindications for TAVI and Killip class ≥ 3.

### MRI protocol

All MRI examinations were performed on a 1.5-T clinical MR imaging unit (AVANTO_fit; Siemens Healthineers AG).

For aortic root sizing and determination of coronary ostial distances, a non-contrast-enhanced, navigator-gated free-breathing 3D whole heart acquisition was conducted using a steady-state free precession (SSFP)-based sequence. The applied imaging parameters were published in detail previously [5].

For determination of the minimal diameters of access vessels, an axial free-breathing, non-contrast-enhanced, two-dimensional (2D) QISS-MRA was performed. QISS-MRA uses an electrocardiographically gated initial in-plane saturation pulse to suppress background tissues, followed by a caudally applied tracking saturation pulse to suppress venous signals. After a quiescent inflow interval, a 2D fat-saturated single-shot balanced SSFP acquisition was performed. The quiescent inflow period overlaps with the period of rapid systolic flow, ensuring maximal inflow of unsaturated spins into the section, even in the setting of very slow flow [9]. Since QISS-MRA is intrinsically directional, the aortic arch had to be scanned twice in order to visualize the ascending and descending arch segment separately, with the tracking pre-saturation pulse on opposite sides, respectively. Imaging parameters are fixed, regardless of the heart rate and other factors, without variation from patient to patient: TR/TE, 3.9/1.74 ms; flip angle, 50°; field of view, 400 × 260 × 3 mm^3^; matrix, 304 (608) × 198 (396); spatial resolution, 1.3 (0.66) × 1.3 (0.66) × 3.0 mm^3^; phase partial Fourier factor, 5/8; GRAPPA imaging factor, 2; bandwidth, 660 Hz/pixel, with fat suppression. The scan length ranged from supraaortic branches to the groins.

### Computed tomography angiography

CTA was performed on a 128-slice dual-source CT (128 mm × 0.6 mm detector collimation, 0.28 s gantry rotation time) and high-pitch factor (3.2; Somatom Definition Flash, Siemens Healthineers AG) with prospective electrocardiographic synchronization to the diastolic phase of the heart. A body weight–adapted injected bolus of 60 to 140 mL of nonionic iodine contrast agent was applied with 370 mg/mL iodine concentration (Iopromide, Ultravist 370, Bayer Pharma AG). The scan length ranged from supraaortic branches to the groins.

### Image interpretation

The aortic annulus (area, perimeter, and diameters) and coronary ostia heights were measured by two blinded radiologists on non-contrast 3D whole heart MRI and on CTA images. MR whole heart images were reconstructed using the multi-planar reconstruction tool, being part of our routine diagnostic reporting software (IMPAX EE workstation, Agfa HealthCare Ges.m.b.H.), as described before [5].

Minimal diameters of the ascending and descending aorta, the abdominal aorta, the left and right common iliac arteries, the left and right external iliac arteries, and the left and right common femoral arteries were measured on QISS-MRA and CTA images. Iliac kinking angle was defined as the deviation between inflow and outflow directions of the iliac arteries and was measured on maximum intensity projections of both QISS-MRA and CTA aortoiliofemoral datasets.

The overall subjective image quality of the 3D whole heart sequence and QISS-MRA was rated by both readers independently and blinded on a 4-point Likert scale (1 = excellent image quality, 2 = good image quality, 3 = acceptable diagnostic despite impairments by artifacts, 4 = non-diagnostic).

### Statistical analysis

Statistical analysis was conducted using Statistical Package for Social Sciences (SPSS) version 24.0 (IBM AG). Categorical variables are presented as frequencies and corresponding percentages. The Shapiro-Wilk test was applied to test for normal distribution (ND). All results are expressed as mean ± standard deviation (SD) (if ND) or as median with IQR (if not ND). Correlations of continuous variables between CTA and MRI measurements were correlated with Pearson’s or Spearman’s rank test as appropriate. Differences in measurement were evaluated by Bland-Altman plots (mean difference ± 2 SD) and compared with Student’s *t* test for paired or unpaired variables (ND) or Mann-Whitney *U* test or Wilcoxon rank test (not ND). The coefficient of variation was calculated by division of the SD with the mean and reported as percent.

ICC by a two-way mixed model for single measures was used to compare measurements and image quality scores between observers and differences were tested with paired Student’s *t* test and Wilcoxon rank test. Decision for transfemoral access, detection of dissection, and angulation < 90° in the access route were compared with Cohen’s kappa. For all tests, a two-tailed *p* value < 0.05 was considered statistically significant.

## Results

### Patient characteristics

In total, 26 patients completed both MRI and CT examinations, 58% were female, and mean age was 83 ± 5 years. In total, 69% of patients showed impaired renal function (eGFR < 60 ml/min/1.73 m^2^); median eGFR was 47 ml/min/1.73 m^2^ (IQR 41–52). Median volume of applied contrast agent for CTA was 70 ml (IQR 70–80).

Median delay between CTA and CMR was 1 day (IQR 0–2). Patient characteristics are listed in Table 1.

**Table 1:** Patient characteristics

Characteristic	Study population (*n* = 26)
Age (years)	83 ± 5
Female (*n* (%))	15 (57)
Body mass index (kg/m^2^)	24.5 ± 4.6
Body surface area (m^2^)	1.73 ± 0.2
Aortic annulus area (cm^2^)	4.3 ± 0.9
Creatinine (mg/dl)	1.1 (1–1.4)
eGFR (ml/min/1.73 m^2^)	47 (41–52)
eGFR (< 60 ml/min/1.73m^2^) (*n* (%))	18 (69)
NT-pro BNP (ng/l)	2080 (780–5910)
hs-cTnT (ng/l)	24 (17–48)
Echo - LVEF (%)	53 ± 14
Contrast agent CTA (ml)	70 (70–80)

*SD* standard deviation, *IQR* interquartile range, *eGFR* estimated glomerular filtration rate, *NT-pro BNP* N-terminal pro brain natriuretic peptide, *hs-cTnT* high-sensitivity cardiac troponin T, *LVEF* left ventricular ejection fraction, *CTA* computed tomography angiography

### Image data acquisition and quality

Average scan time was 5.2 min (IQR 4–6) for 3D whole heart acquisition with a median subjective image quality of 1 (IQR 1–2) stated by both investigators (ICC = 0.920). QISS-MRA scan time was 9 min (IQR 8.3–9.5) and subjective image quality was 1 (IQR 1–2) for both readers (ICC = 0.926). No statistically significant difference between scorings was present (each *p* = 1). Average subjective image quality of aortoiliofemoral CTA was 1 (IQR 1–1.6), compared with 1 (IQR 1–2) for QISS-MRA (*p* = 0.273). Combined median MR image acquisition time for both sequences was 13.5 min (IQR 13–15.5), compared with 1.7 min (IQR 1.1–3.3) for contrast-enhanced CTA (*p* < 0.0001).

### Aortoiliofemoral diameter and transfemoral access capability

The assessment of minimal vessel diameter for aortoiliofemoral TAVI access showed moderate to strong correlations between QISS-MRA and CTA (*r* = 0.572 for the right external iliac artery to *r* = 0.851 forthe thoracic descending aorta, *p* = 0.002 and *p* ≤ 0.0001, respectively). For the comparison of MRA and CTA measurements, see Table 2.

**Table 2:** Comparison of QISS-MRA and CTA measurements of the aortoiliofemoral access route

	QISS-MRA	CTA	Correlations		Bland-Altman plot				
			*r*	*p* value	Bias	LLoA	ULoA	CoV (%)	*p* value
Diameter Ao. asc. (mm)	36.6 ± 4.4	36.5 ± 4.1	0.842	*≤ 0.0001*	0.09	− 4.59	4.77	6.6	0.848
Diameter Ao. desc. thor. (mm)	26 ± 3.4	25.9 ± 3.2	0.851	*≤ 0.0001*	0.11	− 3.39	3.62	6.9	0.790
Diameter Ao. infrarenalis (mm)	15.7 ± 2.5	14.9 ± 2.3	0.786	*≤ 0.0001*	0.73	− 2.38	3.85	10.4	*0.020*
Diameter A. iliaca comm. right (mm)	10 (9.5–11.6)	9.3 ± 2	0.785	*≤ 0.0001*	0.79	− 1.25	2.83	10.6	*0.002*
Diameter A. iliaca ext. right (mm)	8.3 (7–9)	7.4 ± 1.6	0.572	*0.002*	0.44	− 3.01	3.90	23.1	*0.041*
Diameter A. fem. comm. right (mm)	8 (7.8–8.8)	7.3 ± 1.7	0.751	*≤ 0.0001*	0.66	− 1.03	2.34	11.2	*0.002*
Diameter A. iliaca comm. left (mm)	9.7 ± 1.5	8.7 ± 1.4	0.732	*≤ 0.0001*	0.98	− 1.07	3.03	11.3	*≤ 0.0001*
Diameter A. iliaca ext. left (mm)	8 (7–9)	8 (7–9)	0.720	*≤ 0.0001*	0.21	− 1.70	2.13	12.3	0.190
Diameter A. fem. comm. left (mm)	8.2 ± 1.3	7.8 ± 1.5	0.826	*≤ 0.0001*	0.37	− 1.22	1.96	10.2	*0.034*

*p* values set in italics represent statistically significant values (*p* < 0.05)

*MRA* magnetic resonance angiography, *CTA* computed tomography angiography, *LLoA* lower level of agreement, *ULoA* upper level of agreement, *CoV* coefficient of variance

**Fig. 1:**
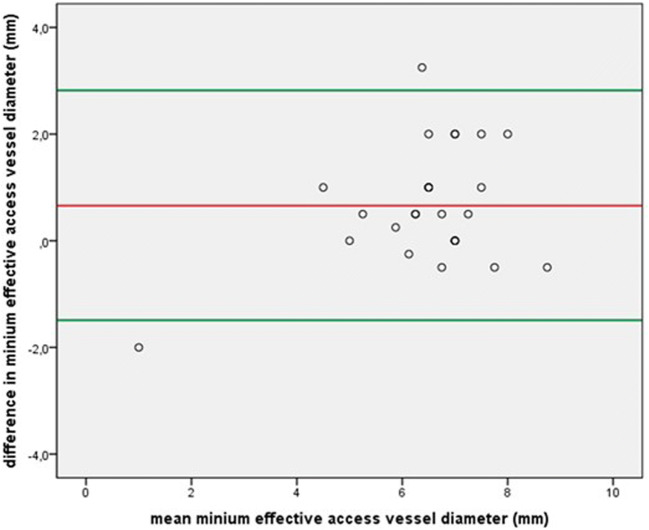
Bland-Altman plot for differences in minimum access vessel diameter between QISS-MRA and CTA. The red line on the Bland-Altman graph represents the mean, the upper green line represents + 2 standard deviations (SD), and the lower green line represents − 2 SD

Thoracic aortic diameters revealed no statistically significant bias between the modalities (*p* = 0.320 to 0.848). Although linear correlations between minimal abdominopelvic access vessel diameters were strong (*r* = 0.572 to 0.851, *p* = 0.002 to ≤ 0.0001), statistically significant differences between QISS-MRA and CTA measurements were found for the infrarenal aorta (bias 0.73 mm, *p* = 0.020), the right and left common iliac arteries (bias = 0.79 mm, *p* = 0.002 and bias = 0.98 mm, *p* ≤ 0.0001 respectively), the right external iliac artery (bias 0.44 mm, *p* = 0.041), and the right and left common femoral arteries (bias = 0.66 mm, *p* = 0.002 and bias = 0.37 mm, *p* = 0.034, respectively), as reported in Table 3. Mean effective minimal vessel diameter between the modalities (7 mm (IQR 6.4–8) vs. 6 mm (IQR 5.7–7) for MRA and CTA, respectively) showed moderate correlation (*r* = 0.521, *p* = 0.006). Bias between the modalities was 0.66 mm (upper and lower limits of agreement: 2.82 mm and − 1.49 mm, *p* = 0.004). The CoV of effective minimal aortoiliofemoral vessel diameter between QISS-MRA and CTA was 10.3% (see Bland-Altman plot, Fig. 1).

**Table 3:** Comparison of 3D “whole heart” MRI and CTA measurements of the aortic root

	MRI	CTA	Correlations		Bland-Altman plot				
			*r*	*p* value	Bias	LLoA	ULoA	CoV (%)	*p* value
Annulus diameter minimum (mm)	19.3 ± 2.7	19.5 (17–22.1)	0.809	*≤ 0.0001*	− 0.63	− 6.28	5.02	14.65	0.334
Annulus diameter maximum (mm)	26.9 ± 4.4	27.8 ± 3	0.679	*≤ 0.0001*	− 0.89	− 7.08	5.3	11.56	0.165
Annulus area (mm^2^)	425.1 ± 86.1	441.6 ± 86.1	0.843	*≤ 0.0001*	− 16.15	− 109.05	76.75	10.93	0.095
Annulus perimeter (mm)	76.5 ± 7.7	78.5 ± 7.7	0.770	*≤ 0.0001*	− 1.93	− 11.94	8.09	6.60	0.066
Ostial height RCA (mm)	13.7 ± 3.1	14.2 ± 2.7	0.887	*≤ 0.0001*	− 0.46	− 3.26	2.33	10.27	0.075
Ostial height LM (mm)	13 ± 2.1	13.2 ± 2.5	0.790	*≤ 0.0001*	− 0.16	− 3.15	2.82	11.63	0.583
Diameter sinus valsalva (mm)	32.6 ± 3.2	32.7 ± 3.4	0.882	*≤ 0.0001*	− 0.17	− 3.30	2.97	4.92	0.634
Diameter ST junction (mm)	25.5 ± 4.2	26 ± 4.2	0.833	*≤ 0.0001*	− 0.46	− 5.11	4.18	9.23	0.320

*p* values set in italics represent statistically significant values (*p* < 0.05)

*MRI* magnetic resonance imaging, *CTA* computed tomography angiography, *LLoA* lower level of agreement, *ULoA* upper level of agreement, *CoV* coefficient of variance, *RCA* right coronary artery, *LM* left main artery, *ST* sinotubular

Decision against transfemoral accessibility was made if bilateral dissection or angulation of iliacofemoral vessels of < 90° or a minimum aorto-biiliofemoral vessel diameter of < 5 mm was found. Bilateral iliac angulation of < 90° was found in 2 (8%) patients by both QISS-MRA and CTA. Consistently, iliofemoral dissection was found unilaterally in 3 (12%) patients by both CTA and QISS-MRA. However, one dissection in the right common femoral artery as delineated by QISS-MRA remained incomprehensible in CTA.

**Fig. 2:**
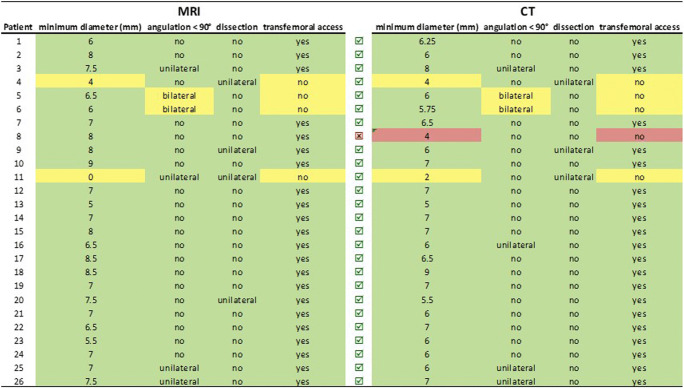
Comparison of hypothetical transfemoral accessibility based on the following decision makers: minimal aortoiliofemoral vessel diameter of < 5 mm, dissection of aortoiliofemoral vessels, angulation of iliac vessels < 90°. Concordant decisions for transfemoral accessibility per patient are indicated by the green boxes. The yellow boxes illustrate cases in which both MRA and CTA show one or more findings that speak against a transfemoral approach. The red boxes indicate the only inter-method mismatch case, based on a minimal vessel diameter of < 5 mm as measured by CTA, whereas MRA revealed a minimal vessel diameter > 5 mm in this case. A green check box indicates concordant decision for or against transfemoral access between QISS and CTA whereas a divergent decision is marked by a red check box

Based on these findings, as depicted in Fig. 2, transfemoral accessibility according to CTA was found in 21 patients (81%) and in 22 patients (85%) according to QISS-MRA (*κ* = 0.866, *p* ≤ 0.0001).

**Fig. 3:**
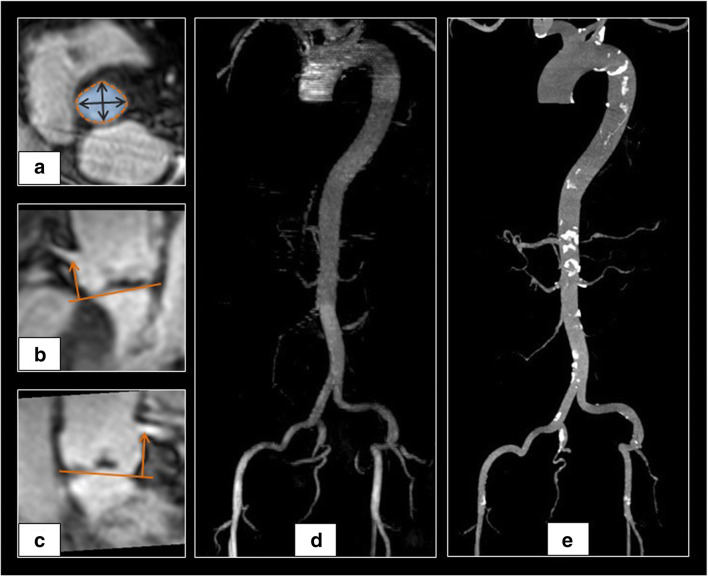
Imaging example of non-contrast 3D “whole heart” MRI for (**a**) aortic annulus measurements at the hinge point plane, the (**b**) right coronary artery, and the (**c**) left main artery ostial height measurement in the longitudinal axis at a right angle to the hinge point plane. Representative maximum intensity projection of aortoiliofemoral QISS-MRA (**d**) and CTA (**e**) datasets

**Fig. 4:**
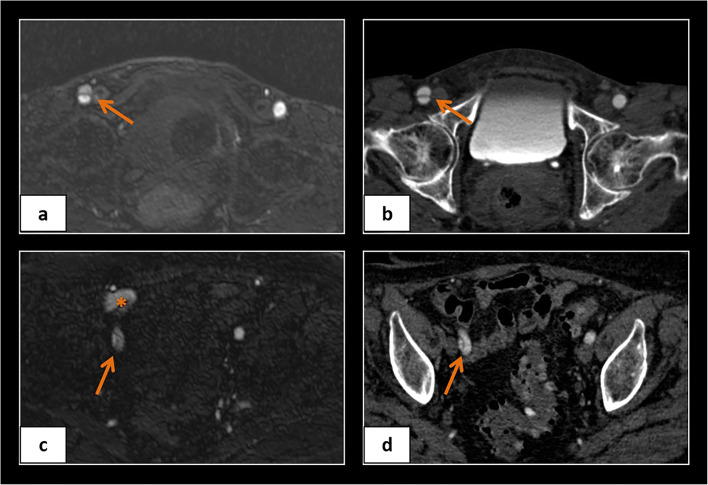
Examples showing dissection of the right common femoral artery in QISS-MRA (**a**) and CTA (**b**) and of the right external iliac artery in QISS-MRA (**c**) and CTA (**d**). The asterisk in (**c**) indicates artifacts due to intestinal gas

Inter-observer reproducibility was high for QISS-MRA, ranking between ICC = 0.862 for the minimal vessel diameter of the left common femoral artery and an ICC of 0.989 for the minimal ascending aorta diameter (all *p* ≤ 0.0001) (Figs. 3, 4). Differences between observers were significant for the ascending aorta (*p* = 0.017) and the left common iliac artery (*p* = 0.027). Also, see Table 4.

**Table 4:** Inter-observer agreement and differences in QISS-MRA measurements of aortoiliofemoral access vessels

Access vessel	Observer 1	Observer 2	*r*	*p* value	Bias	*p* value
Diameter Ao. asc. (mm)	36.7 ± 4.4	36.3 ± 4.6	0.989	*≤ 0.0001*	0.37	*0.017*
Diameter Ao. desc. thor. (mm)	25.9 ± 3.5	25.7 ± 3.1	0.878	*≤ 0.0001*	0.26	0.342
Diameter Ao. infrarenalis (mm)	15.9 ± 2.5	15.8 ± 2.5	0.986	*≤ 0.0001*	0.06	0.571
Diameter A. iliaca comm. right (mm)	10 (9–11)	10 (8.5–12)	0.981	*≤ 0.0001*	− 0.01	0.892
Diameter A. iliaca ext. right (mm)	8 (7–9)	8 (7–9)	0.967	*≤ 0.0001*	0.13	0.519
Diameter A. fem. comm. right (mm)	8 (8–9)	8 (7.5–9)	0.906	*≤ 0.0001*	0.07	0.771
Diameter A. iliaca comm. left (mm)	10 (9–11)	9.4 ± 1.4	0.918	*≤ 0.0001*	0.27	*0.027*
Diameter A. iliaca ext. left (mm)	8 (7–9)	8 (7–9)	0.878	*≤ 0.0001*	0.13	0.334
Diameter A. fem. comm. left (mm)	8 (7–9)	8 (7–9)	0.862	*≤ 0.0001*	0.20	0.201

*p* values set in italics represent statistically significant values (*p* < 0.05)

### Aortic root dimensions

Measurements of the aortic annulus area and perimeter provided strong correlations between non-contrast 3D whole heart MRI and CTA (area: *r* = 0.843; perimeter: *r* = 0.770; both *p* < 0.0001). Mean effective annulus area was similar for MRI and CTA (425 ± 86 mm^2^ vs. 442 ± 86 mm^2^), resulting in a bias of 16 mm^2^ (lower and upper levels of agreement − 109 mm^2^ and 77 mm^2^, respectively, *p* = 0.095). The coefficient of variation was 10.9%. The left and right coronary ostial heights correlated strongly between MRI and CTA (*r* = 0.790 and *r* = 0.887, all *p* ≤ 0.0001, respectively). The difference between MRI and CTA measurements for ostial heights was not significant (LM: bias − 0.2 mm, LLoA − 3.2 mm, ULoA 2.8 mm, *p* = 0.583; RCA: bias − 0.5 mm, LLoA − 3.3 mm, ULoA 2.3 mm, *p* = 0.075). Aortic root measurements are summarized in Table 3.

## Discussion

For a successful TAVI procedure, it is crucial to accurately size the valve prosthesis dictated by aortic annulus dimensions as well as to evaluate access capability of aortoiliofemoral vessels, usually driven by a contrast-enhanced CTA. However, in patients undergoing TAVI, the reported prevalence of acute kidney injury ranges between 8.3 and 41% with an associated increase in 30-day mortality, 1-year mortality, and length of hospital stay [2, 3]. In line with published observations, patients with CKD (eGFR < 60 ml/min/1.73 m^2^) constituted 69% of the overall population of the present study [13]. Moreover, patients with chronic and acute kidney injury, especially at Kidney Disease Outcomes Quality Initiative (KDOQI) stages 4 and 5, are at higher risk to suffer NSF after administration of gadolinium-based MR contrast agents [6]. However, the rapidly growing interest in more sophisticated, non-contrast MR imaging is also driven by reports of intra- and extracranial gadolinium depositions with currently unclear clinical significance [14].

Regarding contrast-free imaging in the context of TAVI, Renker and colleagues presented a rapid, non-contrast, free-breathing, self-navigated three-dimensional MR sequence to study aortic root and vascular access route [15]. However, this feasibility study was conducted in 10 healthy volunteers only. Our MRI evaluation of aortoiliofemoral access routes in 26 patients referred for TAVI planning was based on QISS-MRA measurements. This unenhanced MRA technique was first introduced in 2010 by Edelman et al and until now has been evaluated in a large field of clinical applications at 1.5 T and 3 T [16–19], mainly for the detection and characterization of lower extremity arterial disease [20–24], and also in the demonstration of the pulmonary embolism [19], for coronary imaging [25], and for intracranial MRA [26].

So far, the only study integrating the QISS technique for the assessment of vascular access anatomy showed technical feasibility for TAVI planning but was clearly limited by a very small sample size (5 patients and 10 healthy volunteers) [27]. Our comparatively larger cohort of 26 patients significantly expands these findings by presenting excellent inter-method agreement for the decision of transfemoral TAVI accessibility between QISS-MRA and CTA measurements. Of note, while minimal vessel diameters throughout the access routes correlated strongly between QISS-MRA and CTA measurements, the inter-method bias of minimal infrarenal aorta and iliofemoral vessel diameters was, although in the size range of up to only 1 mm, statistically significant. This observation can be related to several studies reporting limited image quality in the abdominopelvic region owing to motion artifacts as well as flow-related artifacts based on high systolic and diastolic flow velocities and multiple directions of flow [10, 16, 23, 28]. QISS-MRA in general is relatively insensitive to patient motion due to 2D acquisition in the transverse plane. However, the overall longer acquisition time compared with that of CTA and scanning of the pelvic segment at the end of the examination when patient motion is usually observed may render this station more prone to motion artifacts. Moreover, remaining immobile in supine position for an extended period can be challenging for TAVI candidates that are often multimorbid and high-aged. Similar to CTA, the scan direction of QISS-MRA was perpendicular to the mainly vertically oriented descending aorta. Transverse plane imaging of the anatomically more curved, relatively narrow pelvic axis may decrease accuracy in quantification of both anterior-posterior and left-right diameters and may therefore be another explanation for the significant bias in this scan region.

A recent study specifically addressed QISS imaging of abdominopelvic arteries and presented two prototype free-breathing fast low-angle shot-based (FLASH) sequences at 3 T [29]. FLASH QISS-MRA provided improved image quality and sensitivity for detection of stenosis > 50% when compared with SSFP-based QISS-MRA, while specificity was comparable. Especially in patients with metallic implants or artifacts by gas-filled bowel loops, image quality improved with slightly longer image acquisition time of the FLASH QISS technique.

In contrast with our study, where we compared arterial diameters for TAVI planning between QISS and CT angiography, former QISS studies evaluating lower extremity PAD did not quantify artery diameters and considered dichotomous stenosis grading (< 50% and ≥ 50% stenosis) only [9, 10, 16, 17, 22, 30]. Regarding dissecting flaps, both observers reliably detected three unilateral iliacofemoral dissections in both modalities. QISS-MRA provided sufficient arterial signal in both the true and the false lumens where flow is expected to be particularly slow. This is in concordance to the consistent and robust QISS signal behavior across a wide range of flow velocities raging from approximately 5 to > 50 cm/s as shown in a phantom setting [9]. However, QISS-MRA described a single additional dissection of the common femoral artery without evidence of a corresponding CTA correlate. It has to be mentioned that the intraluminal QISS signal, due to its slightly granular aspect, may simulate subtle linear signal attenuations, which requires a QISS-trained eye to avoid false positive dissection findings. However, in general, QISS-MRA showed an excellent subjective image quality. Of note, QISS-MRA lacks visualization of calcified plaque burden since it depicts exclusively the vessel lumen and not the wall. However, calcification in CTA, particularly in small vessels, results in blooming artifact and partial volume averaging with underestimation of the minimal luminal diameter [31]. This may explain in part the significant bias regarding the mean effective minimal vessel diameter between the modalities showing a larger diameter for MRA than CTA.

Moreover, the single inter-method mismatch regarding transfemoral TAVI accessibility in this study may be owed to this problem and is in line with previous reports demonstrating superior performance of QISS-MRA over CTA in heavily calcified vessels [22].

In accordance with a previously published study by our group [5], this study again confirmed high reproducibility of aortic root parameters combined with very low inter-observer variability evaluated by 3D whole heart MR. Strong correlations between CTA and MRI measurements regarding diastolic aortic annulus dimensions are in good concordance with previous studies using this sequence [32].

Comparable prosthesis sizing between CTA and MRA in combination with sufficient access route evaluation highlights the feasibility of this comprehensive unenhanced MRI protocol for pre-procedural TAVI imaging, resulting in reduction of both total radiation dose and amount of applied contrast media. Acute kidney injury (AKI) induced by iodinated contrast media has been shown to mostly occur in the course of pre-procedural TAVI imaging, increasing in-hospital mortality [33]. Especially in chronic kidney disease, in our study concerning 69% of all subjects, as a risk factor for AKI, patients may benefit from contrast-free TAVI imaging since injected contrast medium volumes more than 100 ml and repeated injection of iodinated contrast media within a short interval are associated with an additional increase in AKI risk [34].

As a limitation of the study, the relatively small patient cohort has to be addressed; therefore, more patients referred for TAVR evaluation should be included in future studies.

## Conclusion

This study presents an easy-to-implement contrast-free MRI approach for aortoiliofemoral accessibility evaluation in patients scheduled for TAVI. Comparison with CTA as “gold standard” for minimal vessel diameter showed strong correlations with QISS-MRA measurements, resulting in comparable decisions for transfemoral access capability. The combination of QISS-MRA with an unenhanced 3D sequence for aortic root measurements facilitates TAVI planning without contrast media. Given the high prevalence of renal function impairment in patients referred for TAVI, the presented data highlights this MRI protocol as a reliable, contrast-free guidance alternative.

## Abbreviation

2D: Two-dimensional
3D: Three-dimensional
AKI: Acute kidney injury
CKD: Chronic kidney disease
CTA: Computed tomography angiography
eGFR: Estimated glomerular filtration rate
GRAPPA: Generalized autocalibrating partial parallel acquisition
IBM: International Business Machines
ICC: Intra-class correlation coefficient
IQR: Interquartile range
LLoA: Lower level of agreement
LM: Left main artery
MRA: Magnetic resonance angiography
MRI: Magnetic resonance imaging
ND: Normal distribution
NSF: Nephrogenic systemic fibrosis
PAD: Peripheral artery disease
QISS: Quiescent-interval single-shot
RCA: Right coronary artery
SD: Standard deviation
SPSS: Statistical Package for Social Sciences
SSFP: Steady-state free precession
T: Tesla
TAVI: Transcatheter aortic valve intervention
TE: Echo time
TR: Repetition time
ULoA: Upper level of agreement
